# An Effective 3D Ear Acquisition System

**DOI:** 10.1371/journal.pone.0129439

**Published:** 2015-06-10

**Authors:** Yahui Liu, Guangming Lu, David Zhang

**Affiliations:** 1 Department of Computer Science and Technology, Harbin Institute of Technology Shenzhen Graduate School, Shenzhen, China; 2 Biometrics Research Centre, Department of Computing, The Hong Kong Polytechnic University, Kowloon, Hong Kong; Universitat Pompeu Fabra, SPAIN

## Abstract

The human ear is a new feature in biometrics that has several merits over the more common face, fingerprint and iris biometrics. It can be easily captured from a distance without a fully cooperative subject. Also, the ear has a relatively stable structure that does not change much with the age and facial expressions. In this paper, we present a novel method of 3D ear acquisition system by using triangulation imaging principle, and the experiment results show that this design is efficient and can be used for ear recognition.

## Introduction

Biometric authentication is playing important roles in applications of public security such as access control [1], forensics, and e-banking [2, 3]. In order to meet the needs of different security requirements, new biometrics including palmprint [4], vein [5], ear [6–8] and so on, have been developed. Among them, the ear has been proven to be a stable candidate for biometric authentication due to its desirable properties such as universality, uniqueness and permanence. In addition, an ear possesses several advantages: its structure does not change with age and its shape is not affected by facial expressions [9, 10].

Researchers have developed several approaches for ear recognition from 2D images. Burge and Burger proposed a method based on Voronoi diagrams [8]. They built an adjacency graph from Voronoi diagrams and used a graph matching based algorithm for authentication. Hurley, Nixon and Carter proposed a system based on force field feature extraction [11]. They treated the ear image as an array of mutually attracting particles that act as the source of Gaussian force field. Choras presented a geometrical method of feature extraction from human ear images [12]. Although these approaches show some good results, the performance of 2D ear authentication will always be marred by illuminations and pose variation. Also, the ear has more spatial geometrical information than texture information, but spatial information such as posture, depth, and angle are limited in 2D ear images.

In recent years, 3D techniques have been used in biometric authentication, such as 3D face [13, 14], 3D palmprint [15–17], 3D fingerprint [18, 19] and 3D ear recognition [20–26]. A 3D ear image is robust to imaging conditions and contains surface shape information which is related to the anatomical structure as well as being insensitive to environmental illuminations. Therefore, 3D ear recognition has drawn more and more researchers' attention recently. Yan and Bowyer [20] presented an automated segmentation method by finding ear pit and using an active contour algorithm on both color and depth images, in addition to describing an improved ICP approach for 3D ear shape matching. Chen and Bhanu [21] proposed a 3D ear recognition algorithm based on local surface patch and ICP method. They also presented a 3D ear indexing method [22] which combined feature embedding and a support vector machine based learning technique for ranking the hypotheses. Islam et al. provided an effective method for ear detection using the key points to extract the local feature of the 3D ears [23]. J. Zhou et al. presented a complete 3D ear recognition system combining local and holistic features in a computationally efficient manner [24]. L. Zhang et al. proposed a 3D ear identification scheme based on sparse representation [25]. Even though good results were achieved by the works mentioned above, there is still room for improvement [26].

**Fig 1:**
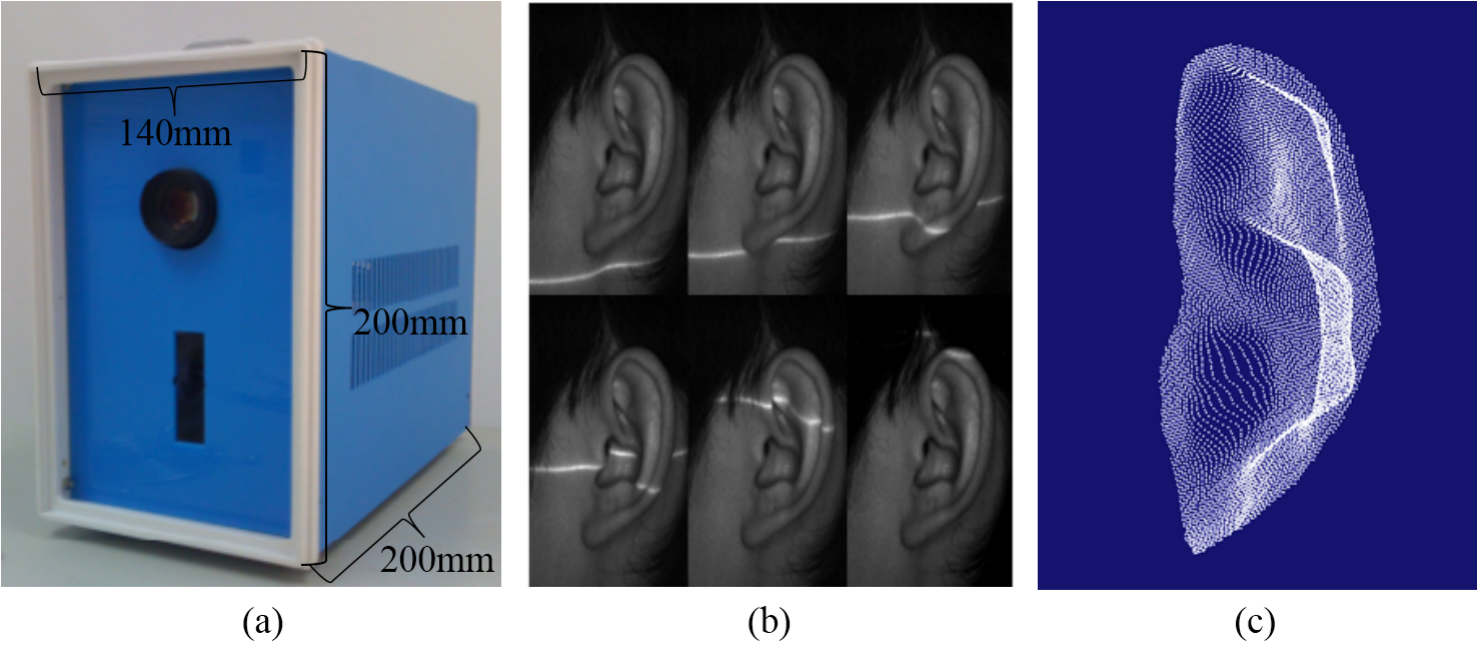
3D ear acquisition system: (a) Our developed 3D ear acquisition device, (b) 2D ear images with laser lines on them, and (c) 3D ear points cloud sample

To date, most of the previous researchers use commercial laser scanners (for example, the widely used Minolta VIVID Series [20–26]) to acquire the 3D ear images. Although these scanners have numerous functions and high accuracy, they are always expensive and big, which is inconvenient for practical biometrics applications. Teresa C.S. Azevedo, João Manuel R.S. Tavares and Mário A.P. Vaz compared two kinds of 3D reconstruction methods: Structure From Motion (SFM) and Generalized Voxel Coloring (GVC) [27], and then proposed a novel volumetric approach to reconstruct and characterize 3D models of human external shapes from 2D images [28]. The approach present high application potential and their results are fruitful, especially for the creation of visually realistic models of humans. But the volumetric methods require an image sequence acquired around the object. Meanwhile these images needed to be calibrated during the 3D model reconstruction. So using this method is not easy to build a real-time device for 3D ear acquisition and recognition. With above considerations, we developed a low-cost 3D ear acquisition system, which is designed specifically for scanning the 3D ear images by using the laser-triangulation principle (as shown in Fig 1 (A)). The main components of our 3D ear acquisition system are: CCD camera, laser projector, step-motor, and motion control circuit. The laser projector projects a red laser line on the object surface. The step motor rotates the laser projector to form a series of scanning lines. The CCD camera is used to record the images in scanning sequence, and transmit them to PC for 3D ear reconstruction and recognition. The system is designed to have the best performance at a reasonable price so that it is suitable for civilian personal identification applications. The dimensions of the device are 140×200×200 (mm), the total scanning and transmission time is less than 2 seconds, and the system can provide the 2D intensity images and 3D points-cloud data for subsequent ear recognition. The 2D and 3D ear images are obtained as shown in Fig 1B and 1C. We collected the 3D samples of ears from 500 individuals using our 3D ear laser scanner. The subjects mainly consisted of volunteers from students and staffs at Shenzhen Graduate School of HIT, including 341 males and 159 females with ages ranging from 20 to 60. These samples were collected on two separate occasions, at an interval of around one month. On each occasion the subject was asked to provide two left side face images. Therefore, each person provided 4 images so that our database contains a total of 2,000 images from 500 different ears. During data collection the subjects sat in a natural position on a chair with a backrest and kept still. The scanner captures the front-view of ear at a distance of about 30cm and an approximately straight-on angle to the side of the face, and the tolerances for pose rotation is ±20°. The subjects were asked to take off all ornaments from their ear and tie their hair back to avoid any occlusions.

The rest of the paper is organized as follows. Section 2 describes the design scheme of our 3D ear acquisition system. Section 3 explains why our system is designed specifically for the ear, and how to optimize the components of the system to make it suitable for ear acquisition. Section 4 shows the performance analysis and comparison. Finally, a conclusion is given in Section 5.

## Design scheme of our acquisition system

### 2.1 Optimized selection of imaging principle

**Fig 2:**
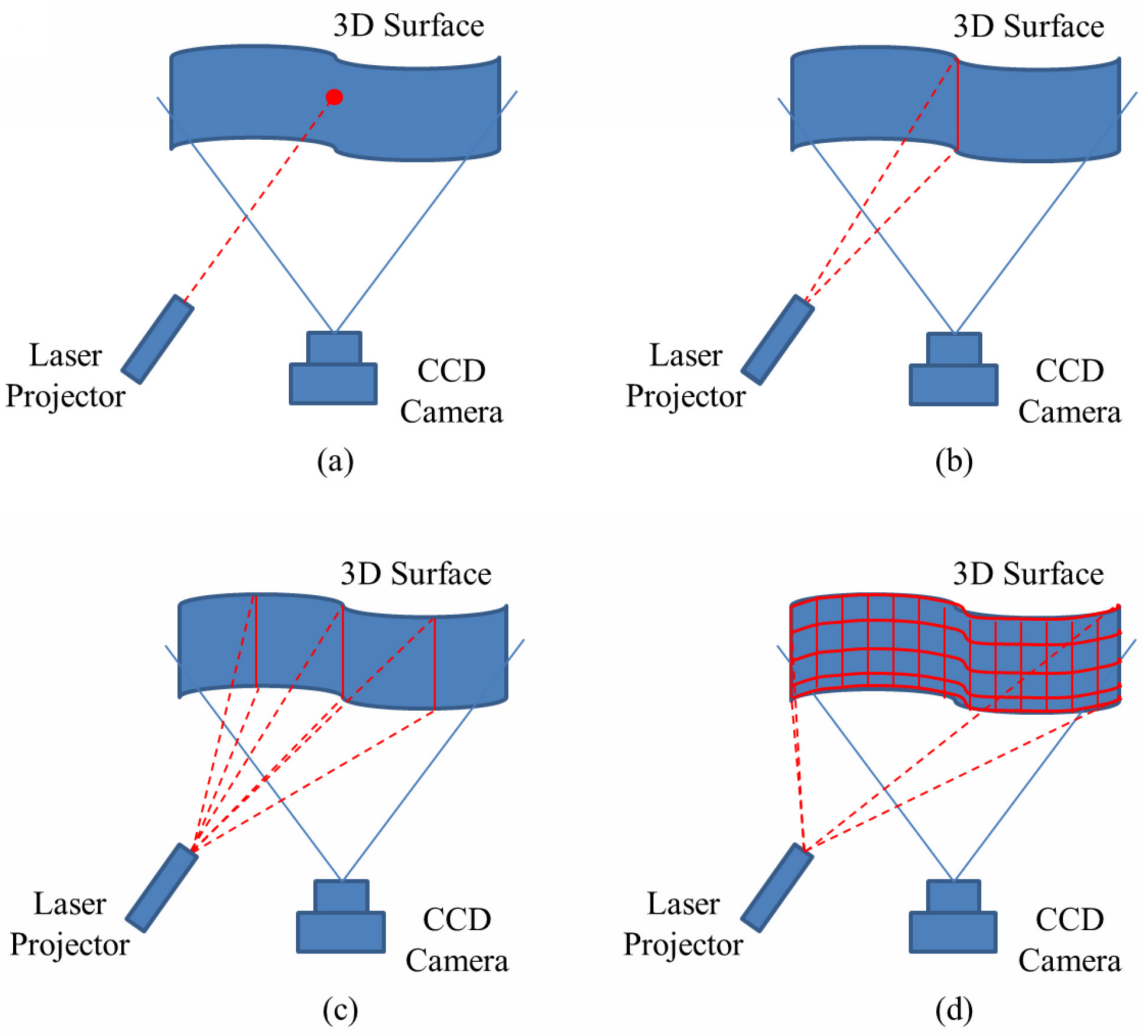
Four types of laser triangulation imaging models. (a) laser point, (b) line scanning, (c) multi-lines scanning and (d) laser grid.

Nowadays, a variety of 3D imaging techniques are available for all kinds of applications. Teresa C.S. Azevedo et al. have given an elaborated discussion in their publications [27, 28]. In the field of biometrics, researchers are usually interested in the following three categories of 3D imaging techniques: multi-view imaging [18, 19], structured light imaging [15,16,17] and laser triangulation imaging [20–25]. Multi-view imaging method is to imitate the human visual system. It has high speed and low cost. Unfortunately, its accuracy of reconstruction depends heavily on the strength of the object features. It is still a challenging task to establish the matching points on smooth surface and low or constant texture [28]. Structured light imaging method uses both projected coded light and sinusoidal fringe techniques. Depth information of the object is encoded into a deformed fringe pattern recorded by a CCD camera. Then the shape is directly decoded from the deformed fringes. This is a fast full field measurement method, and has high accuracy. Although structured light method has the above advantages, its projection unit is usually complex and expensive. E.g. in ref. [15, 17], its projection unit consists of casting lens, LCD panel, controller board, back convergent lens, front convergent lens and white LED light source. Moreover, it is sensitive to ambient light. Laser triangulation imaging employs the well-known triangulation relationship in optics. And it is widely used as its high accuracy and high stability. There are mainly two parts of a laser triangulation imaging system, the image-capturing unit and laser-projection unit. A CCD camera captures the laser light with certain pattern projecting on the object surface. According to the laser light modulated by the surface shape and the triangulation correlation between the projector and camera, the distance from the measured surface to the reference plane can be calculated. Unlike the projection unit of structured light, the laser-projection unit is uncomplicated. There are four kinds of laser triangulation imaging models according to the laser projector patterns: point model, line scanning model, multi-lines scanning model and grid model, as shown in Fig 2. The simplest model is to project a laser point on the surface. It is an easy way to calculate the point position according to the triangulation. However, when it is demanded to measure the coordinates of all points on the entire surface, the point model is not applicable. Because it only measures one point each time, the efficiency is very low. To compensate its inefficiency, the line/multi-lines scanning model and grid model are extended for 3D surface imaging. The line/multi-lines scanning model can obtain the 3D information of objective surface by processing a series of laser scanning images effectively. The grid model usually uses one image with laser grid to reconstruct the 3D surface. Although it is more efficient, its detail is not as good as laser line/multi-lines scanning model. And it is much complex for application. Considering the objective we want to acquire is the partial profile face with ear, we use the single laser line model to establish the system. It is a tradeoff among the cost, complexity and efficiency.

**Fig 3:**
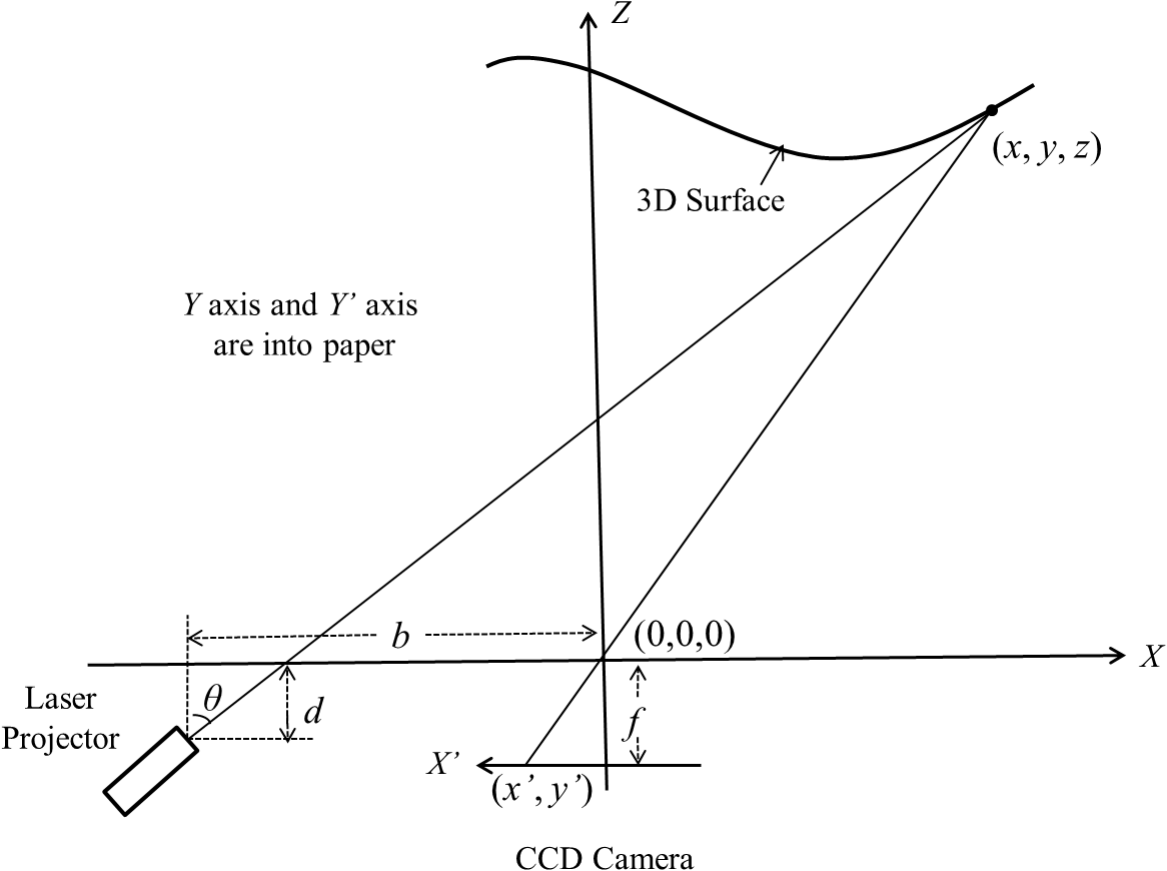
Principle of the laser-triangulation imaging.

Fig 3 illustrates the principle of the laser triangulation imaging. In Fig 3, the reference *X-Y-Z* coordinates is established. The origin of coordinates is the optical center of the camera. And the optical axis is set as *Z* axis. The parameters *b* and *d* are horizontal and vertical offset between the shaft of the laser projector and the origin, while *f* is the focal length of the camera, and *θ* is the projection angle. (*x’*, *y’*) are 2D coordinates of laser line captured by CCD camera. According to the triangulation relationship of these parameters, the 3D coordinates (*x*, *y*, *z*)^T^ can be calculated as following equation:

$$\vec{p}=\left(\begin{array}{c} x\\ y\\ z \end{array}\right)=\left(\begin{array}{c} \frac{x'(b-d\;\text{tan}\;\theta )}{x'+f\;\text{tan}\;\theta }\\ \frac{y'(b-d\;\text{tan}\;\theta )}{x'+f\;\text{tan}\;\theta }\\ \frac{f'(b-d\;\text{tan}\;\theta )}{x'+f\;\text{tan}\;\theta } \end{array}\right)$$ (1)

### 2.2 System framework

**Fig 4:**
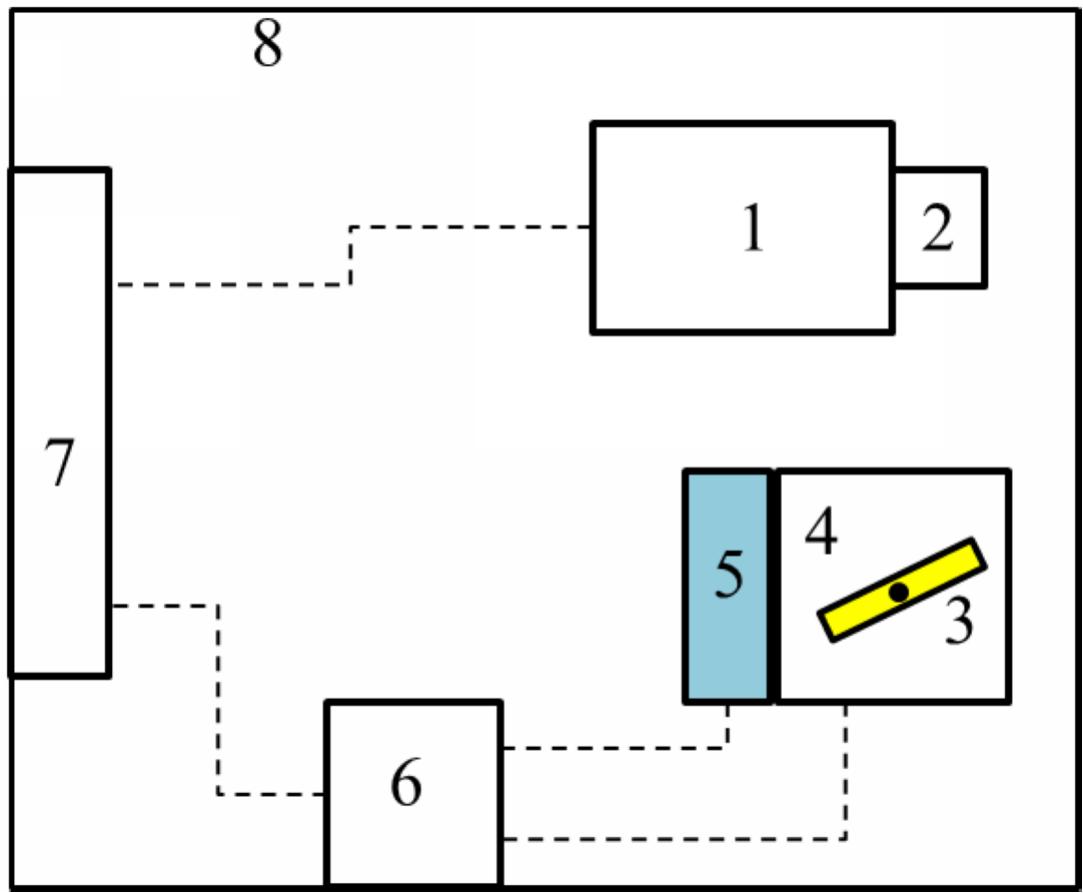
Architecture of the developed 3-D ear data acquisition device. (1) CCD camera, (2) camera lens, (3) laser projector, (4) step motor, (5) photoelectric encoder, (6) motion controller, (7) signal and power control box, and (8) device shell.

Fig 4 illustrates the architecture of the developed 3D ear data acquisition device. The system mainly consists of two parts: the image capturing unit and the projection unit. The image capturing unit contains the CCD camera and camera lens. The projection unit contains the laser projector, step motor, photoelectric encoder, and motion control board. The CCD camera and lens are used to capture the images in scanning sequence. The laser projector projects a red laser line to object surface. The step motor rotates the laser projector to form a series of scanning lines. The photoelectric encoder gives a feedback of rotated position of step motor. And motion controller is in charge of step motor and photoelectric encoder together. It sends command to drive the step motor rotating in a certain angle and direction, at the same time it reads the feedback information from photoelectric encoder. All these components are connected to the signal and power control box to communicate with the computer.

**Fig 5:**
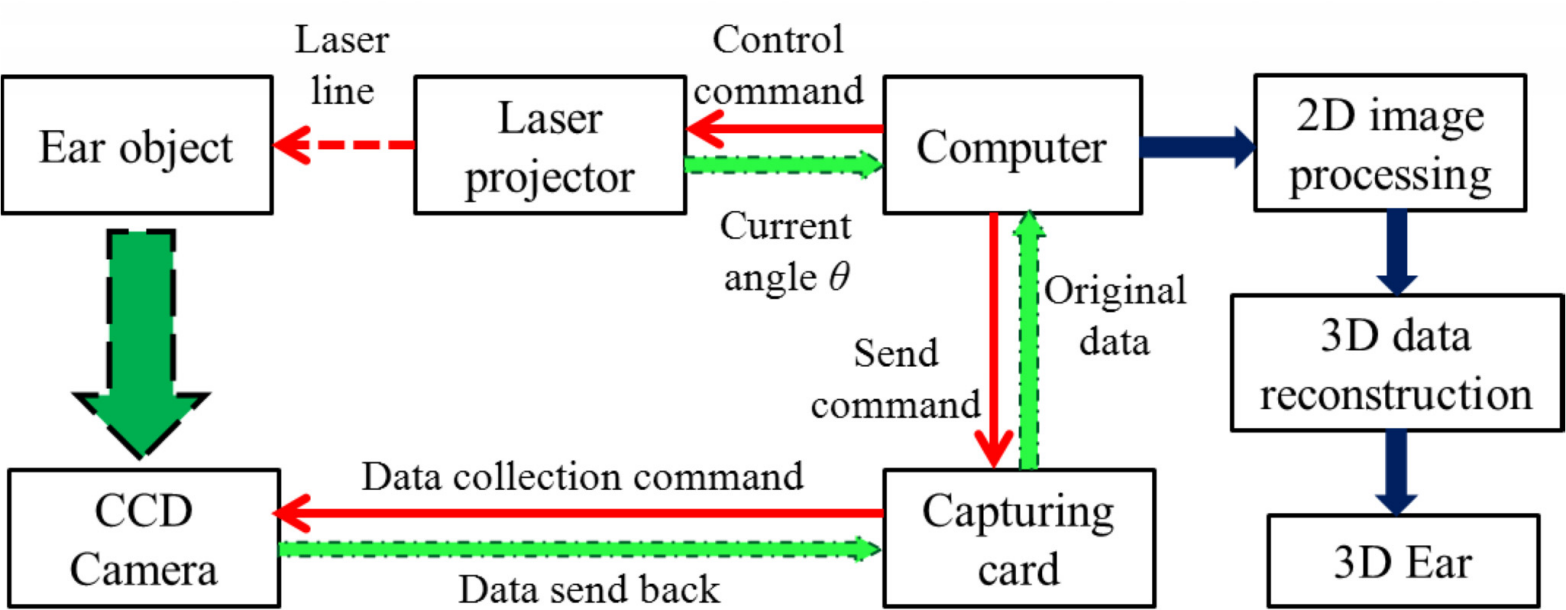
Three-Dimensional ear data collection and processing process (the red solid arrows denote the sending command; the green arrows, from “CCD camera / Laser projector” to “Computer”, denote the data transmission of collecting; and the blue arrows, from “Computer” to “3D Ear”, denote data processing).

The 3D ear data collection and processing process are as follows (Fig 5):

1. The computer send a command to the control circuit, and the motion controller drives the step motor rotate in a preset speed and direction. The motor puts the projector in motion at the same speed and direction, and projects laser lines on the captured ear;
2. At the same time as the laser lines projected on the ear, the computer sends a command via the image capturing card and enables the CCD camera to capture the ear images with laser lines on them. Also their corresponding projection angle values are obtained from photoelectric encoder;
3. Then, a series of 2D ear images with laser lines and angle information are sent back to the computer;
4. Afterwards, we extract the laser lines in each 2D image and obtain a series of 2D coordinates of the laser lines points;
5. Using the laser-triangulation principle the 2D coordinates are translated into 3D coordinates, and the 3D image of the ear is generated.

### 2.3 System calibration

**Fig 6:**
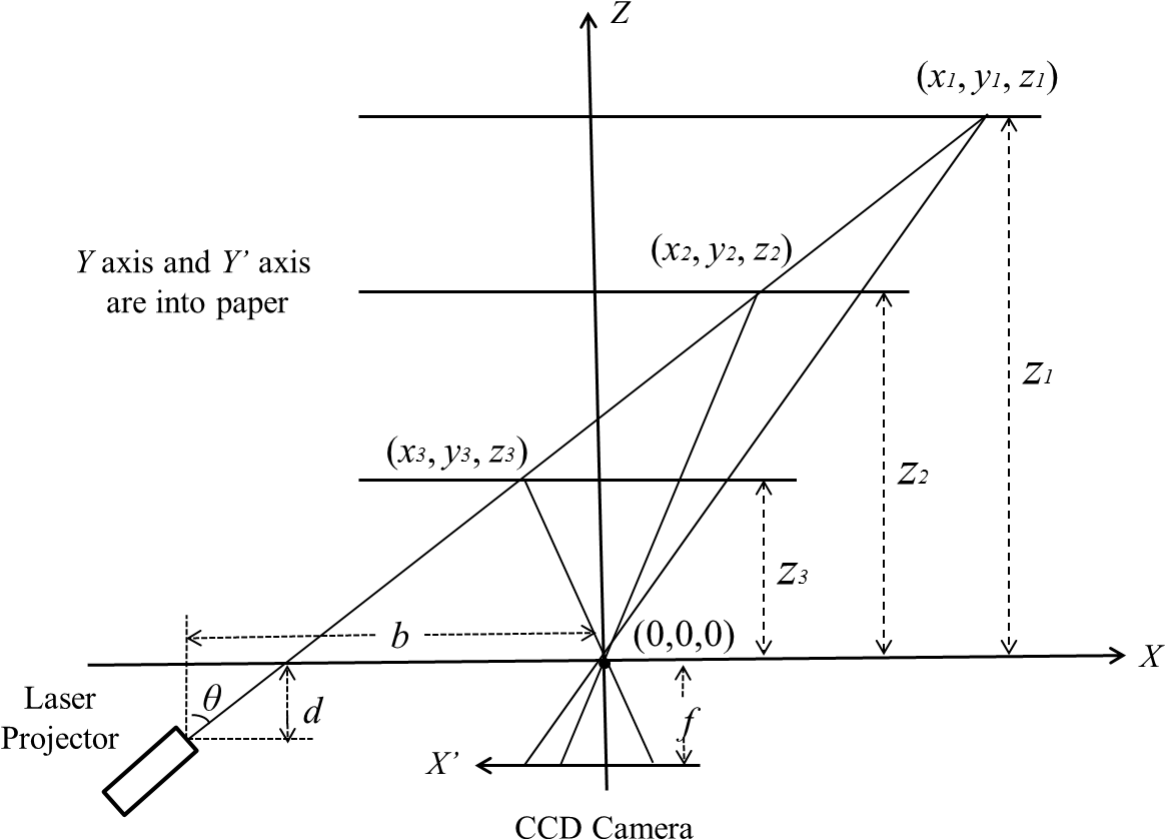
Illustration of the parameters for calibration.

Calibration is an important step for 3D object measurement. Fig 6 shows the principle of the laser-triangulation imaging and the calibration model we used in our acquisition system. This is a simplified model based on the precondition of pinhole imaging. The advantage of this model is that the whole system can be simplified, so that the computational time can be reduced during the reconstruction. The disadvantage of this model is that the lens distortion is not taken into account, which might lead accuracy loss. Nonetheless, we can use two steps to reduce the adverse impact as possible. Firstly, the camera we used is industry standard which has a manual iris and high quality lens. In this way, we can adjust the aperture of the lens as small as possible to approximate the pinhole model which has immunity to lens distortion. Secondly, a comprehensive calibration method was used to solve the non-linear equations and find out the most optimized parameters. There are three parameters *b*, *d* and *f* which need to be decided by calibration as shown in Fig 6, where *b* and *d* are horizontal and vertical offset between the motor shaft and the camera's optical center, while *f* is the focal length of the camera. According to the Eq (1), we get:

$$z=\frac{f'(b-d\;\text{tan}\;\theta )}{x'+f\;\text{tan}\;\theta }$$ (2)

We can see that if *z*, *x’* and *θ* are known, the unknown parameters will be *b*, *d* and *f*. The *x’* can be extracted and calculated by the captured images, and the projection angle *θ* can be read from the photoelectric encoder. Hence, given three different distances *z*_*1*_, *z*_*2*_, *z*_*3*_, as shown in Fig 6, we obtain three equations from Eq 2, and the unknown parameters *b*, *d* and *f* can be resolved.

## Components optimization

Before the components optimization, a brief discussion is given to explain why our acquisition system aimed at 3D ear rather than any other biometrics instances such as 3D fingerprint, 3D palmprint and 3D face. Differed from fingerprint and palmprint, human ear and face have little texture or line feature, but rich spatial geometric features [13–26]. When the 3D models are used for recognition, the fingerprint/palmprint samples should be captured detailedly and precisely so that the texture and line features can be recorded for further processing and matching. This is a difficult task for laser scanning because: if the laser line is bright, there would be a diffuse reflection in the epidermis, which leads to a wider line reflecting to the camera, and the precision would be lost; if the laser line is not bright enough, there would be a loss of details. So the accuracy of laser scanning for skin cannot be very high, unless using the laser projector and the camera of extremely high performances as well as the prices. On the other hand, the accuracy requirements of the 3D ear/face acquisition are much lower than that of the 3D fingerprint/palmprint acquisition. Because people often give more attention to geometrical features than to the texture/line features in 3D face/ear recognition. This allows a tradeoff between accuracy and the cost in 3D ear/face acquisition system development. Furthermore, another consideration of developing the 3D acquisition system for biometrics application is the scanning speed. Under the same spatial resolution, the scanning region is much greater for 3D face than for 3D ear, thus the time and storage consuming would be much greater as well. From the above, we can see that the 3D ear acquisition system is the most promising candidate for achieving a practical 3D biometrics application with high speed suitable accuracy, and low cost. Therefore, the system we developed is focused on 3D ear acquisition. Rooted on this purpose, the system is optimized as follows.

**Fig 7:**
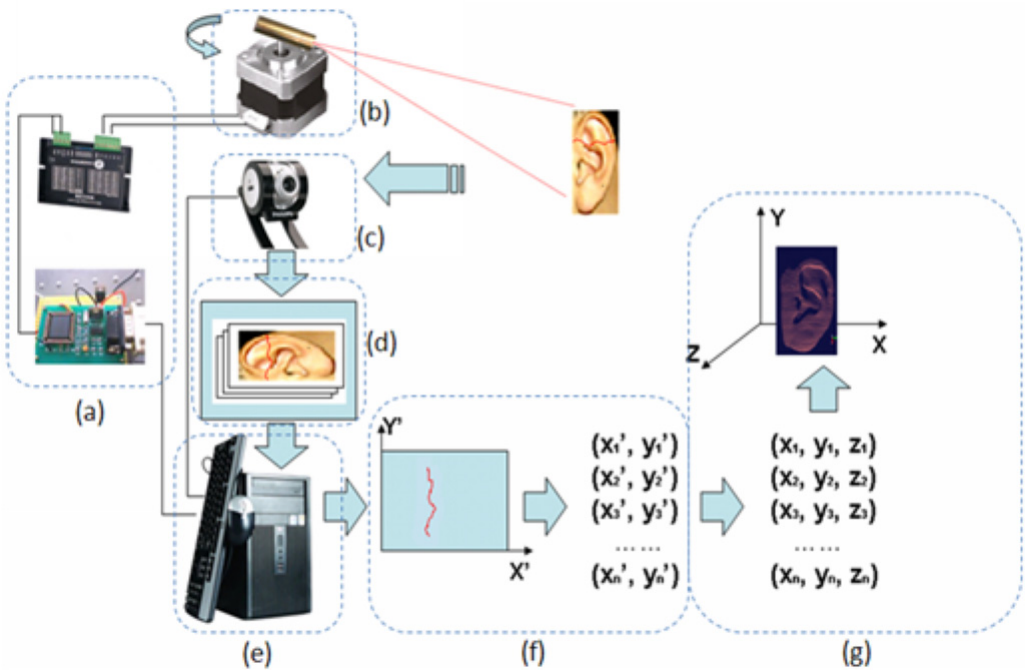
Components of the 3D ear acqusition system.

As we discussed in section II, the core components of the acquisition device are the CCD camera (Fig 7(C)) and projection unit (Fig 7(A) and 7(B)). From Eq (1), we see that the 3D coordinates depend on the 2D coordinates and projection angle *θ*. The 2D coordinates (*x’*, *y’*) are extracted from the laser lines in 2D images captured by CCD camera. The projection angle *θ* is obtained by photoelectric encoder via the motor control board. So the selection of the core hardware is very important for system accuracy and speed.

In our system we concern three important factors of the CCD camera, the resolution, signal noise ratio (SNR), and frame rate, because that the resolution and the signal noise ratio affect the measurement accuracy, and the frame rate determines the scanning speed. Theoretically, the higher these parameters performing, the better performance a system can achieve. Since one of our purposes of developing the 3D scanner is to lower the cost, we prefer to use the standard industrial camera within the acceptable price. The standard resolution for an industrial camera is 768×576 pixels, the SNR is from 50 to 60 dB, and the optional frame rate is from 15 to 120 fps. As Li W. et al discussed in [17], with the growth of SNR, the system accuracy was improved. Therefore we select the camera with SNR 60dB. During the scanning time, there are hundreds of images need to be captured in real-time. So we choose the camera with highest frame rate in order to reduce the time consuming. Thus a CCD camera of 768×576 pixels (resolution), 60dB (SNR), and120 fps (frame rate), is finally selected.

**Fig 8:**
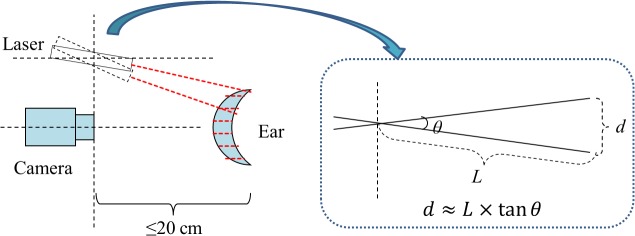
Illustration of one-step rotation in laser scanning.

The projection unit is very important for the scanning stage. Because the performance of the step motor with photoelectric encoder confirms the stability and accuracy of the projection angle *θ*. Generally speaking, the step angle and the subdivision number are two key parameters for a step motor, just like the parameter pulses per revolution (p/r) for the photoelectric encoder. The step angle is the unit angle between two successive positions of a step motor. And subdivision means the motor driver subdivide one step into numbers of micro-steps. For example, if the step angle is 1.8° and the subdivision number is 2, the unit angle for one step will be 0.9°. For most step motor, the step angle is 1.8°. The first three rows of Table 1 illustrate the correlation of step angle and the subdivision number. And the fourth row lists the optional p/r of photoelectric encoder. In the scanning stage, when the projector rotates one step, the scan distance *d* ≈ *L* × tan*θ* (as shown in Fig 8). During our actual scanning, the distance between the projector and human face is less than 20cm. Assume that *L* = 200mm, the *d* value in different subdivision are shown in the fifth row of Table 1. Meanwhile, in the 3D ear acquisition stage, we always scan an area around 15×15cm^2^ to give a certain margin for people putting their ears conveniently. Thus we may estimate the number of images that the camera needs to capture for a complete scanning. The sixth row of Table 1 shows the approximate numbers. According to the imaging principle we know that the larger the number is, the more detailed scanning we can achieve. But too many images would lead to time consuming. So there must be a compromise between fineness and speed. Since the maximum frame rate of our CCD camera is 120 fps, we calculate the capturing time in different subdivision numbers (listed in the bottom row of Table 1). Considering the capturing time, transmission time, storage consumption, and image processing workload, we set 4 as the subdivision number, and choose 1000 p/r for photoelectric encoder. At last, a safe laser projector of security class II is selected to project red laser line. Table 2 lists the core components and their specifications respectively.

**Table 1:** Illustration of parameters variation with different subdivision numbers.

**Subdivision number**	1	2	**4**	8	16	32
**(°/ step)**	1.80	0.90	**0.45**	0.23	0.11	0.06
**(steps/round)**	200	400	**800**	1600	3200	6400
**Encoder (p/r)**	500		**1000**	2000	4000	N/A
***d* (mm)**	6.29	3.14	**1.57**	0.80	0.38	0.21
**2D images number**	24	48	**96**	188	395	715
**Scanning time (s)**	0.2	0.4	**0.8**	1.6	3.3	6.0

**Table 2:** Specifications of core components.

**CCD Camera**	Resolution: 768× 576, SNR: 60dB, Frame rate: 120fps
**Motion unit**	Step angle: 1.8°, Sundivision number: 4, Encoder: 1000 r/p
**Laser projector**	Security class: II, Light traits: red line

## Performance analysis and comparison

In this section, a series of experiments are implemented to analyze the performance of our 3D ear acquisition system. After the calibration and optimization, the depth (z direction) precision of the 3D image measured by this system is ±0.5 mm. Since the main purpose of our system is to capture the 3D ear models for recognition, the image quality is first illustrated intuitively by 3D points-clouds and contour maps of the 3D ears, and then it is analyzed quantitatively by the statistical result of the 3D ears verification based on iterative closest point (ICP) alignment. Finally, a comparison between our 3D ear acquisition system and the commercial laser scanner (VIVID 910) is given.

### 4.1 3D ear data analysis

The source ears consisted of volunteers from students and staffs at Shenzhen Graduate School of Harbin Institute of Technology. We have obtained written consent from the participants prior to study. The study is approved by the Academic Committee of the Department of Computing of Harbin Institute of Technology, Shenzhen Graduate school. One of the responsibilities of the committee is to ensure the research programs are consistent with academic ethics. The research application of 3D ear acquisition has been discussed on meeting by professors and staffs of the committee, and has obtained its written approval signed by the Department Head. Since our research work does not involve the patient or privacy, also all the participants have signed a data collection agreement to give consent to publish the image of their ear for academic purpose, all the data and figures published this paper can be fully available for interested researchers.

**Fig 9:**
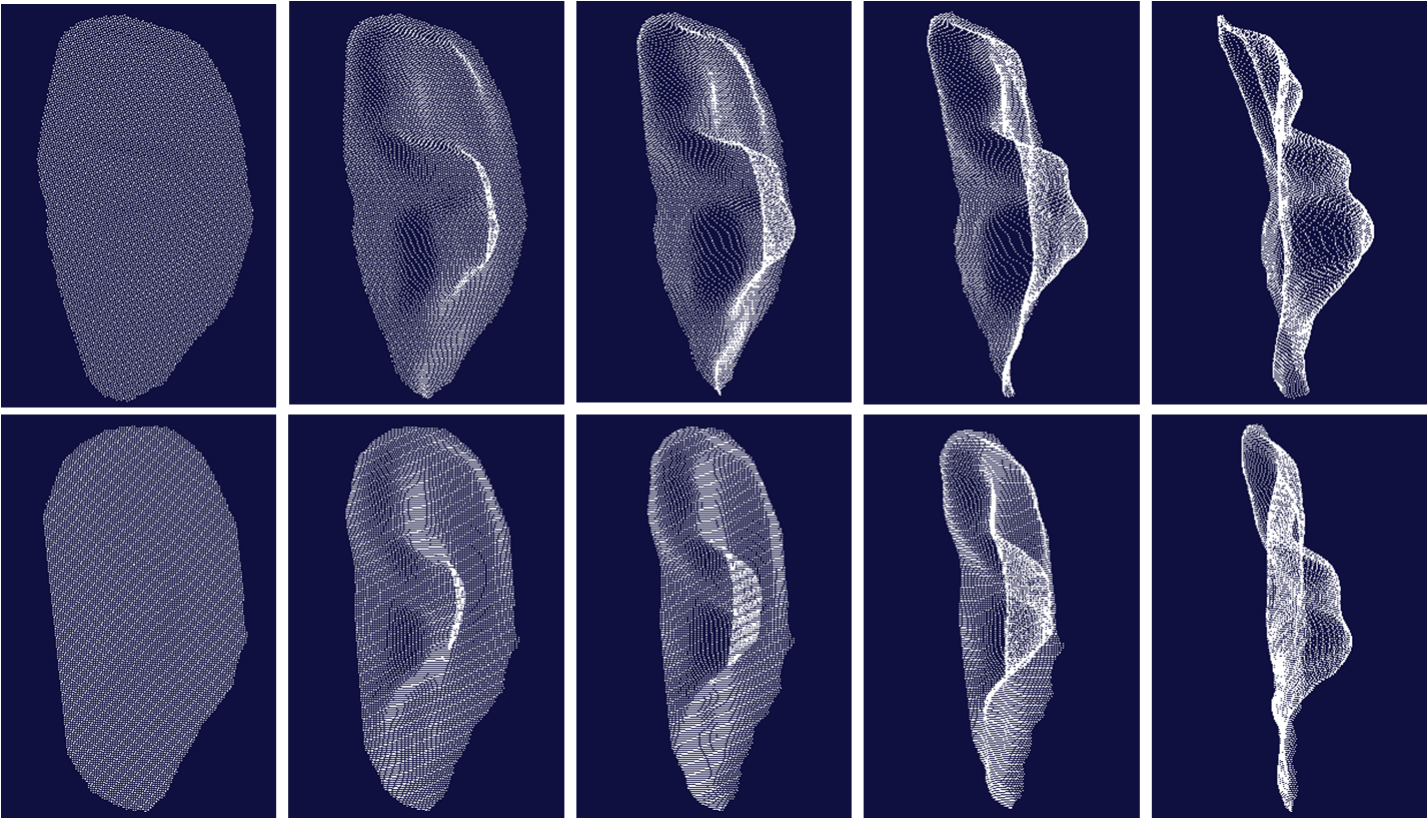
Three-dimensional ear samples viewed in different angle (each row is collected from one ear).

Fig 9 is two typical 3D ear samples captured by our device, where each row is the 3D points cloud from one ear viewed at different angle. We can see the 3D data cloud is smooth and able to display geometric shapes of the 3D ear.

**Fig 10:**
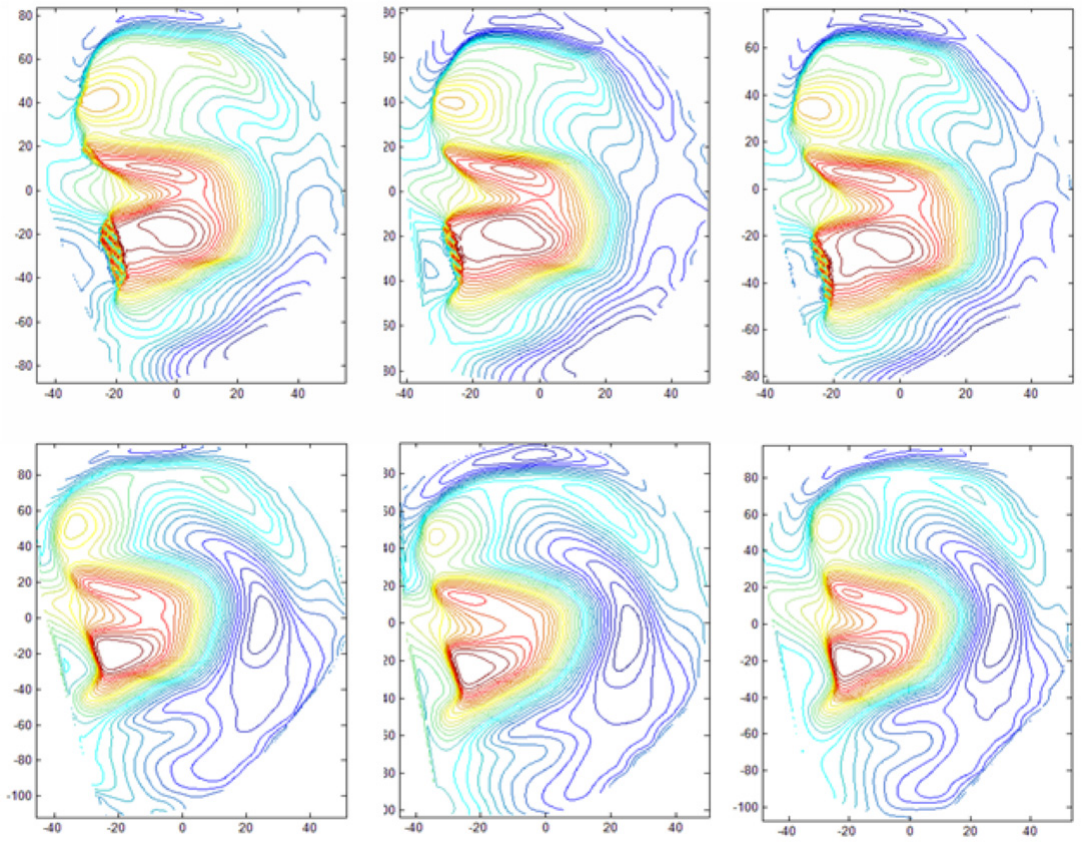
Contour maps of different ear samples (the top row is contour maps drawn from one ear, the bottom row is contour maps drawn from another ear).

In addition, we extract the contour maps of 3D ears' samples to illustrate their geometrical shapes. As shown in Fig 10, the six samples were collected at different sessions from two different ears. The top three contour maps are extracted from one ear, the bottom three contour maps are extracted from another ear. From the contour maps, it can be seen intuitively that the geometrical shapes of 3D ear samples from the same object are stable; on the other hand, for different ears they are distinguishable. Therefore, we can safely assume that the 3D ears' samples captured by our acquisition system would be suitable for 3D ear recognition. In order to corroborating the assumption, a 3D ear database was established by using the developed 3D ear acquisition system. The database contains 2000 samples from 500 volunteers, including 341 males and 159 females. We collected the 3D ears on two separate occasions, at an interval of around one month. On each occasion, the subject was asked to provide two samples. Then the modified ICP alignment is used for samples matching, and the statistical results are illustrated in the form of genuine and impostor distribution curves, as well as the receiver operating characteristic (ROC) curve.

Image registration methodologies have been widely researched and used in numerous fields. Francisco P.M. Oliveira and João Manuel R.S. Tavares provided a state of the art review of these registration methodologies [29]. Among all these methodologies, the ICP algorithm developed by Besl and Mckay [30, 31] is used to register two given points in a common coordinate system. Each iteration of the algorithm calculates the registration by selecting the nearest points. Variants of ICP have been proved to be effective to align 3D ears in previous works [20–23]. Below we explain the algorithm used in our case.

1. Initialization of the rotation matrix *R*_*0*_ and translation vector *T*_*0*_.
2. Given each point in a test ear, find the corresponding point in the model ear.
3. Discard pairs of points which are too far apart using a tolerance distance *t*.
4. Find the rigid transformation (*R*, *T*) and apply it to the test ear.
5. Go to 2) until no more corresponding points can be found or the maximum iteration number is reached.

**Fig 11:**
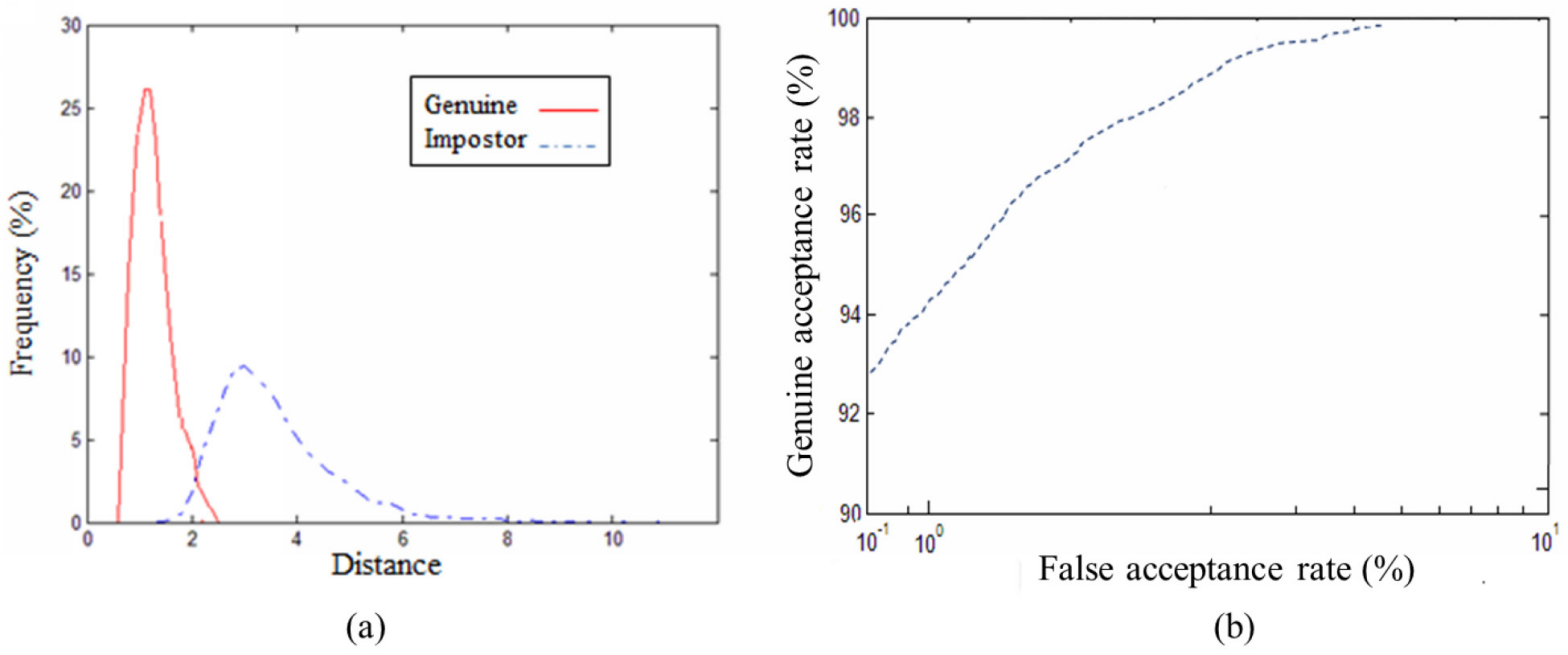
Matching results based on database. (a) Genuine-Impostor distribution curve, (b) ROC curve.

In our experiments we used 10 as the tolerance distance for establishing closest point correspondence, and the maximum iteration number is 50 times. After rotation and translation the average distance of all corresponding points is calculated as the distance of model-test pair. Fig 11 shows the matching results of 3D ear samples in our database, (a) is the distance distribution of genuine and impostor pairs, (b) is the ROC curve. The equal error rate (EER) is 3.54% in our experiment.

### 4.2 Comparison

Table 3 provide a performance comparison with the Minolta Vivid 910 range scanner which is a widely used commercial scanner and has been used to acquire 3D ear data for UND data set [18][29]. The acquisition time refers to the total scanning and transmission time; the resolution refers to the 2D resolution of the output images; accuracy refers to the depth precision of the measurement; dimensions refers to the width, height and length of the scanner; in addition the weight and price are also listed. Although the measurement accuracy of our acquisition system is inferior to that of Vivid 910, the experimental results in section 4.1 prove that this accuracy is capable for 3D ear recognition. Moreover, our 3D ear acquisition system has a higher speed, smaller size, and much lower cost. All these traits make our system suitable for 3D ear authentication in practical biometrics applications.

**Table 3:** Performance Comparison with commercial scanner (Vivid 910).

	Acquisition Time (s)	Resolution (pixels)	Accuracy (mm)	Dimensions (mm)	Weight (kg)	Price (USD)
**Vivid 910**	4	640 × 480	±0.1	213×413×271	11	> 20,000
**Our Scanner**	2	768 × 576	±0.5	140×200×200	3	< 1,000

## Conclusions

This paper presents a novel 3D ear imaging system, which can acquire high quality 3D ear images in real time. The main components of our 3D ear scanner are CCD camera, laser projector, step-motor, photoelectric encoder and motion control circuit. The laser-triangulation principle is used for the 3D image reconstruction. The architecture of the system and its calibration are discussed. The system is designed to have good performance at a reasonable price so that it is suitable for civilian personal identification applications. The experiments show that the developed 3D ear acquisition system can obtain effective 3D information from an ear. At the end, we have constructed a large 3D ear image database which can be used for future feature extraction and performance analysis in later work.
